# Potential use of human adipose mesenchymal stromal cells for intervertebral disc regeneration: a preliminary study on biglycan-deficient murine model of chronic disc degeneration

**DOI:** 10.1186/s13075-014-0457-5

**Published:** 2014-10-08

**Authors:** Giovanni Marfia, Rolando Campanella, Stefania Elena Navone, Ileana Zucca, Alessandro Scotti, Matteo Figini, Clara Di Vito, Giulio Alessandri, Laura Riboni, Eugenio Parati

**Affiliations:** Cerebrovascular Diseases Unit, IRCCS Foundation Neurological Institute “C. Besta”, Milan, Italy; Laboratory of Experimental Neurosurgery and Cell Therapy, Division of Neurosurgery, Fondazione IRCCS Ca’ Granda Ospedale Maggiore Policlinico Milano, University of Milan, Milan, Italy; Unit of Neurotraumatology, Division of Neurosurgery, San Carlo Borromeo Hospital, Milan, Italy; Scientific Direction, IRCCS Foundation Neurological Institute “C. Besta”, Milan, Italy; Department of Medical Biotechnology and Translational Medicine, LITA-Segrate, University of Milan, Milan, Italy

## Abstract

**Introduction:**

Biglycan is an important proteoglycan of the extracellular matrix of intervertebral disc (IVD), and its decrease with aging has been correlated with IVD degeneration. Biglycan deficient (*Bgn*^−/0^) mice lack this protein and undergo spontaneous IVD degeneration with aging, thus representing a valuable *in vivo* model for preliminary studies on therapies for human progressive IVD degeneration. The purpose of the present study was to assess the possible beneficial effects of adipose-derived stromal cells (ADSCs) implants in the *Bgn*^−/0^ mouse model.

**Methods:**

To evaluate ADSC implant efficacy, *Bgn*^−/0^ mice were intradiscally (L1-L2) injected with 8x10^4^ ADSCs at 16 months old, when mice exhibit severe and complete IVD degeneration, evident on both 7Tesla Magnetic Resonance Imaging (7TMRI) and histology. Placebo and ADSCs treated *Bgn*^−/0^ mice were assessed by 7TMRI analysis up to 12 weeks post-transplantation. Mice were then sacrificed and implanted discs were analyzed by histology and immunohistochemistry for the presence of human cells and for the expression of biglycan and aggrecan in the IVD area.

**Results:**

After *in vivo* treatment, 7TMRI revealed evident increase in signal intensity within the discs of mice that received ADSCs, while placebo treatment did not show any variation. Ultrastructural analyses demonstrated that human ADSC survival occurred in the injected discs up to 12 weeks after implant. These cells acquired a positive expression for biglycan, and this proteoglycan was specifically localized in human cells. Moreover, ADSC treatment resulted in a significant increase of aggrecan tissue levels.

**Conclusion:**

Overall, this work demonstrates that ADSC implant into degenerated disc of *Bgn*^−/0^ mice ameliorates disc damage, promotes new expression of biglycan and increased levels of aggrecan. This suggests a potential benefit of ADSC implant in the treatment of chronic degenerative disc disease and prompts further studies in this field.

## Introduction

The degeneration of the intervertebral disc (IVD) is a physio-pathological process accelerating with age and considered as the major cause of diverse spine disorders. Indeed, IVD degeneration represents the main cause of the low-back pain that affects the majority of the adult population, and causes a huge loss of time from work as well as relevant medical expenses [1]. As demonstrated by Furukawa *et al*. [2], the degenerative changes in the IVD that occur in pathologic processes are similar to those associated with normal aging. In addition, IVD degeneration may be premature or early onset of the aging process, or taken as a distinct process from aging [3].

The IVD is the largest enclosed and avascular tissue of the human body, and possesses a peculiar microenvironment, characterized by high mechanical and osmotic pressures, severe hypoxia, and a very limited supply of nutrients [4]. Its core, called the nucleus pulposus (NP), is composed of a jelly-like material that consists mainly of water, as well as the extracellular matrix (ECM) components, including a loose network of collagen fibers and proteoglycans. This ECM composition is similar to the articular cartilage, as it contains several members of the small leucine repeat proteoglycans (SLRPs) family [5]. Temporal and spatial expressions of some SLRPs, in particular biglycan, have been demonstrated in the human IVD [6-8] and their decreasing level appears to correlate with age and degeneration of human IVD [9]. IVD degeneration has been found to spontaneously occur in biglycan-deficient (*Bgn*^*−/0*^) mice, which therefore represent a valuable *in vivo* model to study degenerative disc disease [2]. In this animal model, 7-Tesla magnetic resonance imaging (7TMRI) is a noninvasive tool that is able to evaluate pathological evolution over time and therapeutic follow up [10].

Different therapies have been proposed for the treatment of IVD degeneration, and among them, the use of cell therapy strategies to supplement/replenish the cell population in IVD degeneration appears promising [11-13]. For example, rabbit annulus fibrosus (AF) cells cultured in an atelocollagen honeycomb-shaped scaffold have been allografted into the lacunae of NP of rabbit IVD to treat degeneration through a tissue-engineering method. This approach showed *in vivo* cell proliferation activity with a hyaline-like cartilage production [14].

Our group has recently demonstrated that it is possible to isolate mesenchymal stromal cells from pathological IVD samples, and these cells exhibit stem-cell-like properties [15]. Despite the fact that mesenchymal stromal cells represent a good *in vitro* tool to clarify the mechanisms underlying IVD degeneration, their use in cell therapy for spinal disorders remains unknown, mainly due to difficulty obtaining normal IVD tissue specimens as well as a sufficient number of cells required for therapy. Besides stromal cells derived from IVD tissue, it has been suggested that mesenchymal stromal cells derived from the adipose tissue (ADSCs) could be more accessible and effective cells to treat IVD disease [16,17]. Indeed, ADSCs can be easily isolated from fat tissue of patients with IVD degeneration, and of relevance, they exhibit both proliferative and differentiation capability in appropriate culture conditions [18].

In the present study we assess the possibility to improve disc regeneration by intradiscal injection of ADSCs in the spontaneous and progressive model of IVD degeneration of *Bgn*^−/0^ mice, where IVD changes and degeneration occur due to byglican deficiency [2]. We focused on the efficacy of the cell therapy monitoring treatment effects by high resolution *in vivo* imaging and *ex vivo* histological examination of IVD tissues. We additionally investigated whether ADSC implant increases the production of biglycan and aggrecan in the degenerated IVD.

## Methods

### ADSC isolation and expansion

The study was approved by the local institutional review board of the IRCCS Foundation Neurological Institute “C. Besta” (Milan, Italy), and conformed to the WMA Declaration of Helsinki. Patients’ informed consent to the procedure was obtained. Adipose tissue was collected from peri-umbilical adipose tissue from four healthy male donors (mean age, 45 ± 6 years) undergoing elective abdominal surgery. In particular, abdominal subcutaneous fat specimens of about 1 to 2 cm^3^ (corresponding to 1 to 2 g) were obtained by needle aspiration from the peri-umbilical area, under local anesthesia (1% xylocaine). Once the tissue was aspirated, the sample was placed in a Falcon tube containing Dulbecco’s phosphate-buffered saline (D-PBS) (Euroclone, Milan, Italy) (1:1, w/v) with the addition of penicillin and streptomycin solution (1%) (Sigma-Aldrich, Basel, Switzerland), and sent to the laboratory for tissue processing.

The adipose tissue was extensively washed with D-PBS and after centrifugation (300 g, 12 minutes), the infranatant, containing hematopoietic cells, was removed. The tissue was cut into small pieces with fine scissors (Martin KLS, Tuttlingen, Germany), and then mechanically dissociated using a 1-mL aerosol-resistant tip. Cells were resuspended in 10 mL D-PBS and centrifuged at 123 *g* for 10 minutes. The pellet was resuspended in 500 μL of D-PBS and again mechanically dissociated by a 200-μL aerosol-resistant tip. Cells were resuspended again and centrifuged as described above. Finally, the pellet was resuspended in 10 mL of chemically defined Stem Cells Medium (SCM) [15,19]. The SCM composition consisted of DMEM-F-12 supplemented with 10% FBS (Gibco, Grand Island, NY, USA), 10 ng/mL basic fibroblast growth factor 2 (FGF2) (human recombinant, Peprotech, Rocky Hill, NJ, USA, or Upstate Biotechnology, Lake Placid, NY, USA), and 20 ng/mL epidermal growth factor (EGF) (human recombinant, Sigma-Aldrich, Milan, Italy). Cells were then seeded in tissue culture plates (NUNC, Thermo Scientific Illkirch, Cedex, France) at 1 to 3.5 × 10^3^/cm^2^ density and were maintained in a humidified incubator with 5% CO_2_ at 37°C. After 24 to 48 h from plating, cultures were gently washed with D-PBS to remove unattached cells and then fed with fresh media. When cells achieved about 70% confluence, they were detached from the tissue culture plates using TrypLE Select (Gibco) and plated at a density of 5 × 10^3^ cells/cm^2^. Cultures between passage 3 (P3) and passage 5 (P5) were expanded and used for experimental analyses. The remaining cells were cryopreserved in cryopreservation medium composed of dimethylsulfoxide (Sigma Aldrich) (10% in FBS).

### Flow cytometric immunophenotyping

For each sample, 5 × 10^4^ ADSCs between P3 and P5 were characterized by means of fluorescence-activated cell sorting (FACS). Cells were incubated with appropriate phycoerytrin (PE)- or fluorescein isothiocyanate (FITC)- conjugated monoclonal antibodies to test the expression of a pattern of mesenchymal, hematopoietic, endothelial and immunological markers including: CD14, CD34, CD45, CD73, HLA-DR (BD Pharmingen, San Jose, CA, USA), CD105 (AbDSerotec, Raleigh, NC, USA), CD90 (Millipore Temecula, CA, USA) and CD19 (Beckman Coulter, Cassina de’ Pecchi, Milano, Italy). After 30 minutes at 4°C, cells were washed once with D-PBS, fixed with 4% paraformaldehyde (PFA) (Sigma Aldrich), and analyzed using a FACS scan flow cytometer and Cell Quest software (BD Pharmingen, San Jose, CA, USA). According to the manufacturers’ instructions, specific isotype-matched mouse immunoglobulins (BD Pharmingen) were used as control. At least 2 × 10^4^ events were acquired for each sample. Non-viable cells or cell debris were excluded by physical gating.

### Multipotent differentiation capacity of ADSCs

ADSCs were tested for their capacity to differentiate into adipocytes, chondrocytes and osteocytes according to the minimal criteria suggested by Dominici *et al.* [20]. The procedure was described by Navone *et al*. [21] (*Materials and methods*).

### Experimental animals

Procedures involving animals and their care were conducted in conformity with all procedures following institutional guidelines which, in turn, are in compliance with national (DL number 116, GU Suppl. 40, February 18 1992, Circolare number 8, GU, 14 July 1994) and international laws and policies (EEC Council Directive 86/609, OJ L 358, 1 December 2012, 1987; NIH Guide for the Care and Use of Laboratory Animals, US National Research Council, 1996). The protocol for the use of laboratory animals was approved by the ethical committee of the IRCCS Foundation Neurological Institute “C. Besta” (Milan, Italy), and by the Italian Ministry of Health (number NTN-01-10). *Bgn*^−/0^ mice, generated by gene targeting in embryonic stem cells [22], were kindly provided by Dr Marian Young (National Institutes of Health, Bethesda, MD, USA), Dr Ariane Melchior-Becker and Dr Jens W Fischer (University of Essen, Germany) and Dr Liliana Schaefer (Johann Wolfgang Goethe-University, Frankfurt, Germany). C57Bl6 mice were purchased from Charles River (Calco, Lecco, Italy). We used male mice for experimental animals because the biglycan gene is located on the X chromosome and absent from the Y chromosome. All mice maintained their hybrid (C57Bl6/129) genetic background. DNA was isolated from tail samples, and the genotype of the wild type (WT) and *Bgn*^−/0^ mice was determined by PCR [23]. The successful genetic deletion in *Bgn*^−/0^ mice was previously assessed by the absence of biglycan mRNA and protein [22]. Animals were enrolled at 6 months of age and characterized for disc degeneration until 19 months by 7TMRI (7 T Biospec 70/30 USR, Bruker, Ettlingen, Germany). C57Bl6 (n =10) male mice were considered as WT controls. All animals were supplied with food and water *ad libidum*. Temperature, humidity and night-day cycles were maintained according to the standards set up by the research animal services.

### MRI characterization of the animal model

In order to study the timing of disc degeneration and evaluate the time of cell implantation, *Bgn*^−/0^ mice (n =10) and WT mice (n =10) were monitored every 2 weeks from 6 months to 18 months of age with the same acquisition protocol.

### Preparation of ADSCs for transplant

ADSCs isolated from the four donors satisfied criteria proposed by Dominici *et al*. [20]. Briefly, ADSCs, which are plastic-adherent when maintained in standard culture conditions, expressed CD105, CD73 and CD90, and lacked expression of CD45, CD34, CD14, CD19 and HLA-DR surface molecules. When cultured in a selective culture condition, ADSCs were able to differentiate into osteoblasts, adipocytes and chondroblasts. After four *in vitro* passages, ADSCs derived from one donor were harvested by Tryple Select (Gibco), washed and resuspended in saline solution.

### Procedure of ADSC injection into the IVD in *Bgn*^*−/0*^ mice

At 16 months of age, after confirmation of disc degeneration by 7TMRI, ADSCs or saline were administrated according to the following procedure. General anesthesia, consisting of induction with 5% isoflurane (Aerrane, One Baxter Parkway, Deerfield IL, USA) in 30% oxygen in air at a flow rate of 1 L/minute was given, and anesthesia was maintained with 2.0 ± 0.4% isoflurane. Five minutes after induction of anesthesia, supplemental analgesia was provided by intraperitoneal injection of buprenorphine (Sigma Aldrich) (0.3 mg/kg in saline, 1 mL given over 5 minutes). With the animals (n =10 each for placebo and the ADSC-transplanted group) lying supine, both the abdominal skin and peritoneum were incised. The bowel was displaced to allow an anterior approach to the lumbar vertebral segment. IVD at the level of the first lumbar vertebra (L1) to L2 was exposed and ADSCs or saline treatment was administrated by a Hamilton syringe (using a 33-gauge blunt needle). Cells (8 × 10^4^) or saline were injected 1 mm deep in a final volume of 5 μL in a time of 2 minutes. After injection, the needle was maintained inside the disc for a further 2 minutes to reduce the spilling of the injection solution. The bowel was then repositioned and the wound was closed using 5–0 Nylon suture (Ethicon Inc., Somerville, NJ, USA). Post-intervention animals were left to recover on heated pads and then transferred to their own cages, and monitored daily for 3 months.

### Radiological evaluation

The effectiveness of ADSC transplantation on L1 to L2 discs was evaluated by 7TMRI of the lumbar tract of the spine one day before transplantation, immediately after surgery (30 minutes) and every 14 days until 12 weeks after transplantation. The acquisition protocol was optimized in order to reduce exposure time to anesthesia.

### Acquisition protocol

The experiments were carried out using a high-field 7TMRI (Bruker) scanner equipped with a gradient system reaching the maximum amplitude of 400 mT/m. Mice were anesthetized with 1.5 to 2.0% isoflurane (flow rate 0.8 L/minute) and positioned on an animal bed equipped with a nosecone for gas anesthesia. To detect the depth of anesthesia and the animal health condition during the MRI study, the respiratory rate and temperature were monitored by a pneumatic and rectal sensor, respectively. A 75-mm birdcage linear coil (Rapid Mr International, Columbus, OH, USA) was used for radio frequency excitation and a rat brain surface coil (Rapid) was used for signal reception. Animals were placed supine on the rat brain surface coil, so that the lumbar tract of the spine could be placed at the centre of the coil’s sensitive part. For anatomical references and to test the correct animal alignment in the magnet bore, T_2_-weighted images (T_2_-wi) were acquired in three orthogonal planes: axial, sagittal, and coronal. The acquisition protocol included T_1_- and high-resolution T_2_-wi. T_1_-wi was acquired by spin echo sequence with echo time (TE) 11.720 ms, repetition time (TR) 401.306 ms, slice thickness (ST) 1 mm, in-plane resolution 0.105 mm, number of averages (NA) 6 and high resolution T_2_-wi by a rapid acquisition with relaxation enhancement (RARE) sequence (TR/TE =3000/14 ms, RARE factor =8, slice thickness =0.07 mm, in plane resolution =0.086 mm, NA =8). Both sequences investigated the three orthogonal orientations: axial, coronal and sagittal.

### MRI assessment of IVD regeneration after ADSC transplantation

Once the acquisition protocol had been optimized and implantation time determined, the whole protocol was repeated on mice in the placebo group (n =10) and in the ADSCs group (n =10), every two weeks for three months after implantation; the mouse being placed in the same position for easier inter-scan comparison.

### Post processing

A signal intensity index was determined to assess the degree of disc regeneration after transplantation. The operated discs were identified on the high resolution T_2_-wi and the signal intensity was quantified on the corresponding axial T_1_- and T_2_-wi slice using Paravision software (Paravision 5.1, Bruker). A circular region of interest of 1 mm^2^ was drawn at the center of the disc and in the adjacent spinal cord at every time point. The signal intensity from the spinal cord was used as the intensity reference within the scan and the index was therefore defined by the ratio between the disc and the spinal cord signals; thus the normalized indexes could be compared between different animals at different times [10]. Mean values were computed in the L1 to L2 disc where histological examination detected the presence of implanted cells. The selection of regions of interest was repeated independently by two blinded reviewers and the mean from the two values was considered.

### Histological confirmation

Animals were sacrificed at 12 weeks after ADSC injection. The L1 to L4 vertebral bodies were entirely fixed in 4% PFA containing 0.1 M sodium phosphate buffer (Euroclone). After 3 weeks of decalcification with RapidCal Immuno (BBC Biochemical, Mount Vernon, WA, USA), the tissues were embedded in Optimal Cutting Temperature (OCT) compound (Sakura Finetek, Torrance, CA, USA) and sectioned to 20 μm thickness. The sections were stained with Safranin-O (Sigma Aldrich) and counterstained with human specific anti-HuNu antibody (MAB 1281, Chemicon Millipore, Billerica, MA, USA) (1:100) to identify human cells. The histological grading system for disc degeneration devised by Masuda [24] was used, focusing on the morphological changes in the IVD structure. Each sample was interpreted by two blinded histologists. The histological evidence of disc regeneration was interpreted considering the recovery of tissue matrix and IVD structure [25].

### Immunohistochemistry

The disc sections were incubated overnight at 4°C with the appropriate primary antibody against Human Nuclei (HuNu), biglycan, aggrecan, Ki67 and Iba1, diluted in 1% bovine serum albumin (BSA) in D-PBS. Sections were then washed in D-PBS containing 0.1% BSA and incubated 1 h at room temperature (RT) with a secondary antibody conjugated with a fluorochrome (Molecular Probes). Sections were then washed and mounted in Vectashield H-1000 (Vector Laboratories, Peterborough, UK). The following primary antibodies were used: mouse anti-HuNu (1:100 dilution; Chemicon,), goat anti-human biglycan (1:250; Abcam, Cambridge, UK), goat anti-human aggrecan (10 μg/mL; R&D Systems, Minneapolis, MN, USA), rabbit anti-human Ki67 (1:200; Chemicon), rabbit anti-mouse Iba1 (1 μg/mL; Wako Chemicals GmbH, Neuss, Germany). Nuclear staining (1:5000; 4',6-diamidino-2-phenylindole (DAPI), (Invitrogen, Life Technology, Monza, Italy) was applied and used to quantify the total number of cells. Immunohistochemistry acquisitions were performed by two blinded observers.

### Cell count

Images of cells positive to HuNu (as a marker of injected ADSCs), and to biglycan and aggrecan (as markers of *in vivo* differentiation) were acquired by microscopy (Leica TCS SP2 AOBS, Leica Microsystems, Milan, Italy) after specific immunocytochemical staining at 3 months after transplantation. The quantification of positive cells was performed by Axion Vision Software (Carl Zeiss Microscopy GmbH, München Germany). Briefly, cell counts were performed on a minimum of nine independent fields of photomicrographs captured with 40X objective. Total counts of each marker’s immunoreactive cells were performed, and the number of positive cells per culture was expressed as percentage of total cells and of HuNu-positive cells.

### Statistical analysis

The intensity index variation over time was evaluated by SPSS software (IBM). Repeated-measures analysis of variance (ANOVA) was performed pairwise at 3, 5 and 12 weeks post surgery with respect to baseline and between the groups at the same week. *P*-values lower than 0.05 were considered statistically significant. For each cell-treated animal, we estimated the correlation between intensity index and number of ADSCs found in the disc at the histological exam.

## Results

### ADSC culture and phenotypic characterization

ADSCs were isolated from human adipose tissue sample obtained from healthy donors. After seeding in the used culture conditions, isolated ADSCs grew as adherent monolayer and presented a fusiform, fibroblastic-like morphology.

**Figure 1:**
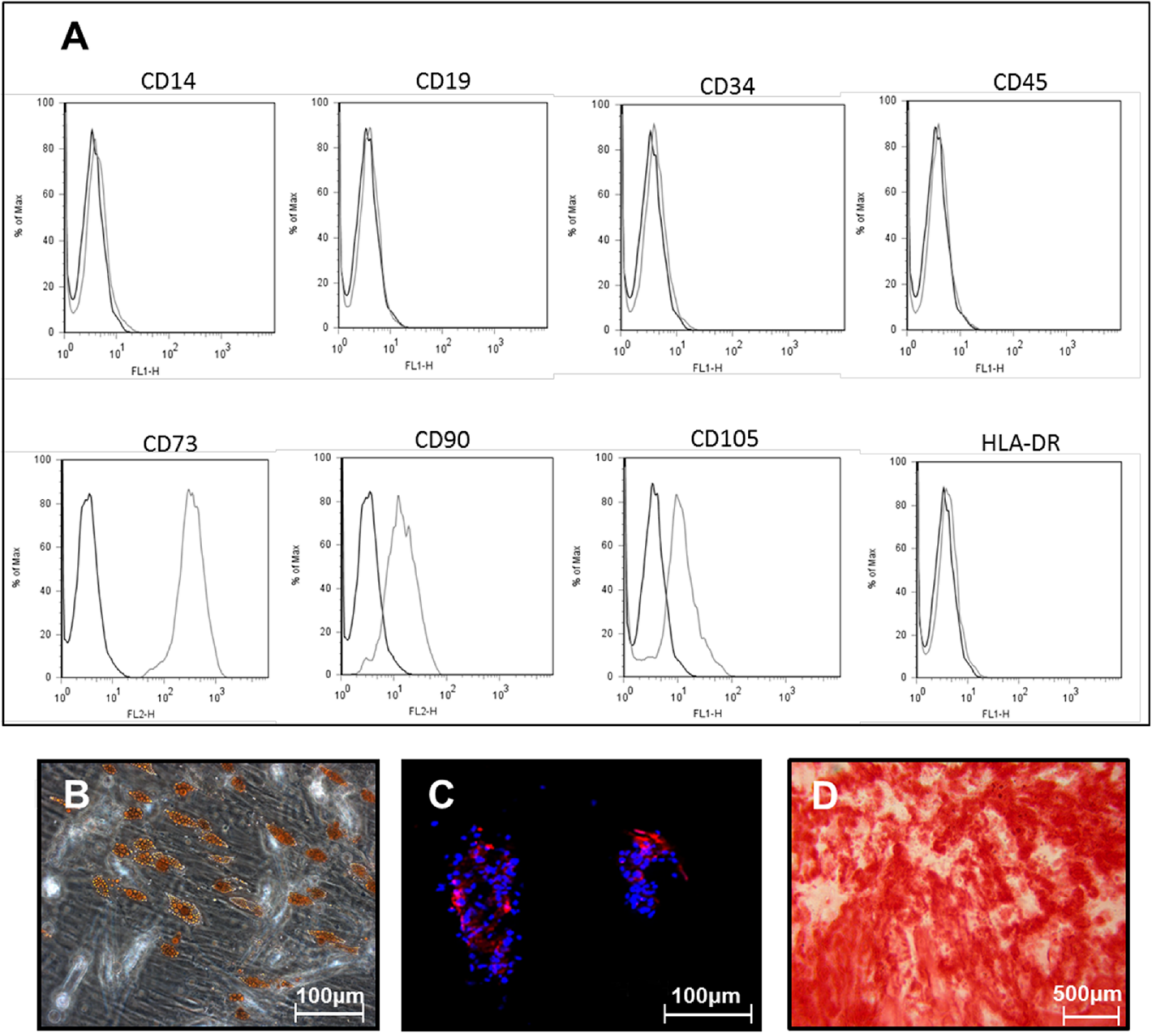
**Features and immunophenotypic mesenchymal profile of human-derived adipose mesenchymal stem cell (ADSCs). (A)** Immunophenotypic mesenchymal profile of ADSCs shows that these cells express a high percentage of positivity for CD73, CD90, CD105, and a low-positivity fraction for CD14, CD19, CD34, CD45 and HLA-DR. After selective stimulation, ADSCs exhibited adipogenic, chondrogenic and osteogenic potential, which was demonstrated by Oil Red O staining of lipid droplets **(B)**, aggrecan deposition **(C)**, and Alizarin Red stained calcium nodules **(D)**.

Immunophenotypic FACS analysis of multiple surface epitopes showed that ≥90% ADSCs expressed mesenchymal stem cell (MSC) markers, including CD73, CD90, and CD105 (Figure 1A). In contrast, less than 2% of ADSCs expressed the hematopoietic cell markers CD14, CD34, and CD45, and the immunological markers CD19 and HLA-DR. Moreover, when cultured in selective media, ADSCs exhibited tripotent differentiation capacity, being able to differentiate into adipogenic, chondrogenic and osteogenic cells (Figure 1B,C,D).

### Identification of degeneration and regeneration from 7TMRI findings

**Figure 2:**
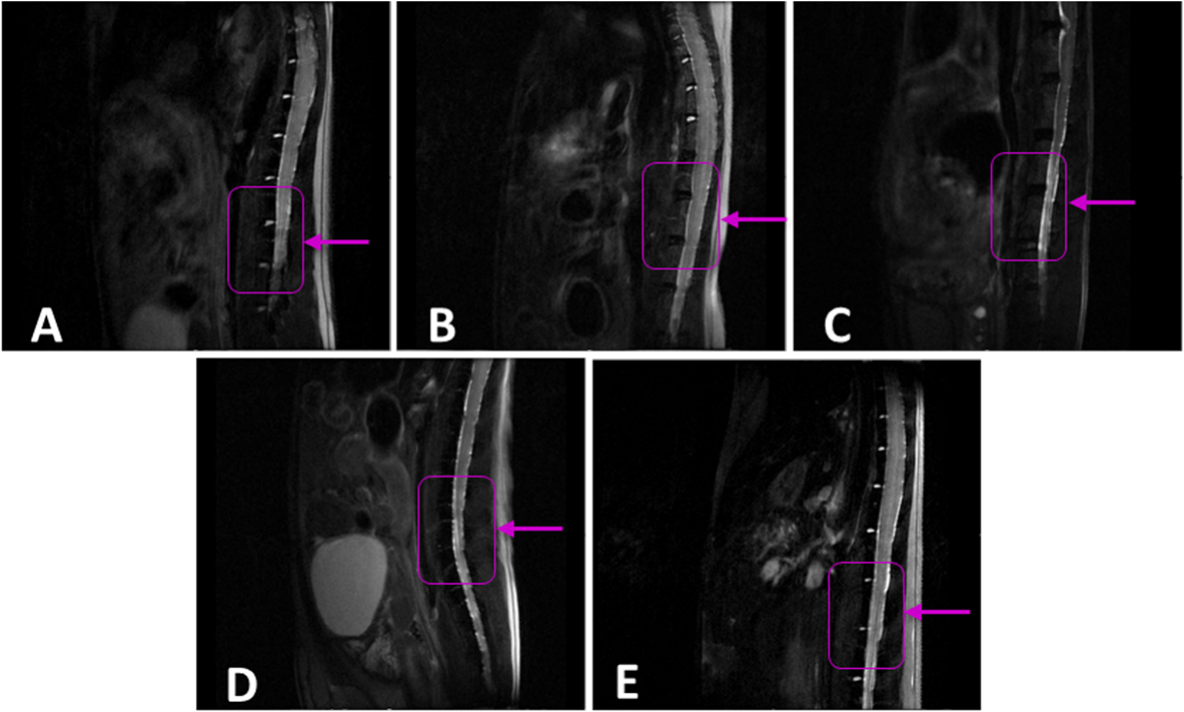
**Magnetic resonance imaging characterization of the intervertebral disc (IVD) of biglycan-deficient (** ***Bgn*** ^**−/0**^ **) and wild type (WT) mice over time.** T_2_-weighted images of the *Bgn* ^−/0^ mouse IVD were acquired at months **(A)** 6, **(B)** 11, **(C)** 16 and **(D)** 18, and of WT mice IVD at month 16 of life **(E)**. Boxes and arrows identify the spinal column level at vertebrae L1 to L2.

Figure 2 shows sagittal T_2_-wi in an IVD *Bgn*^−/0^ mouse over time at 6, 11, 16 and 18 months of life (Figure 2A,B,C,D, respectively). These MRI analyses of the *Bgn*^−/0^ mice revealed disc degeneration as a hypointense signal, opposite to the usual bright signal in T_2_-wi of the WT disc. The degeneration process was already observed at 11 months and reached maximal degeneration at 16 to 18 months, resulting in a complete hypointense signal, which has been shown to be related to the loss of IVD proteoglycans and water content [26]. Based on these results, using a ventral retroperitoneal approach at the level of L1 to L2, *Bgn*^−/0^ mice were implanted with ADSCs at 16 months, when IVD degeneration was maximal. Of note, the signal of IVDs in the WT mice strain (C57Bl6) at this age did not show any intervertebral alteration (Figure 2E).

**Figure 3:**
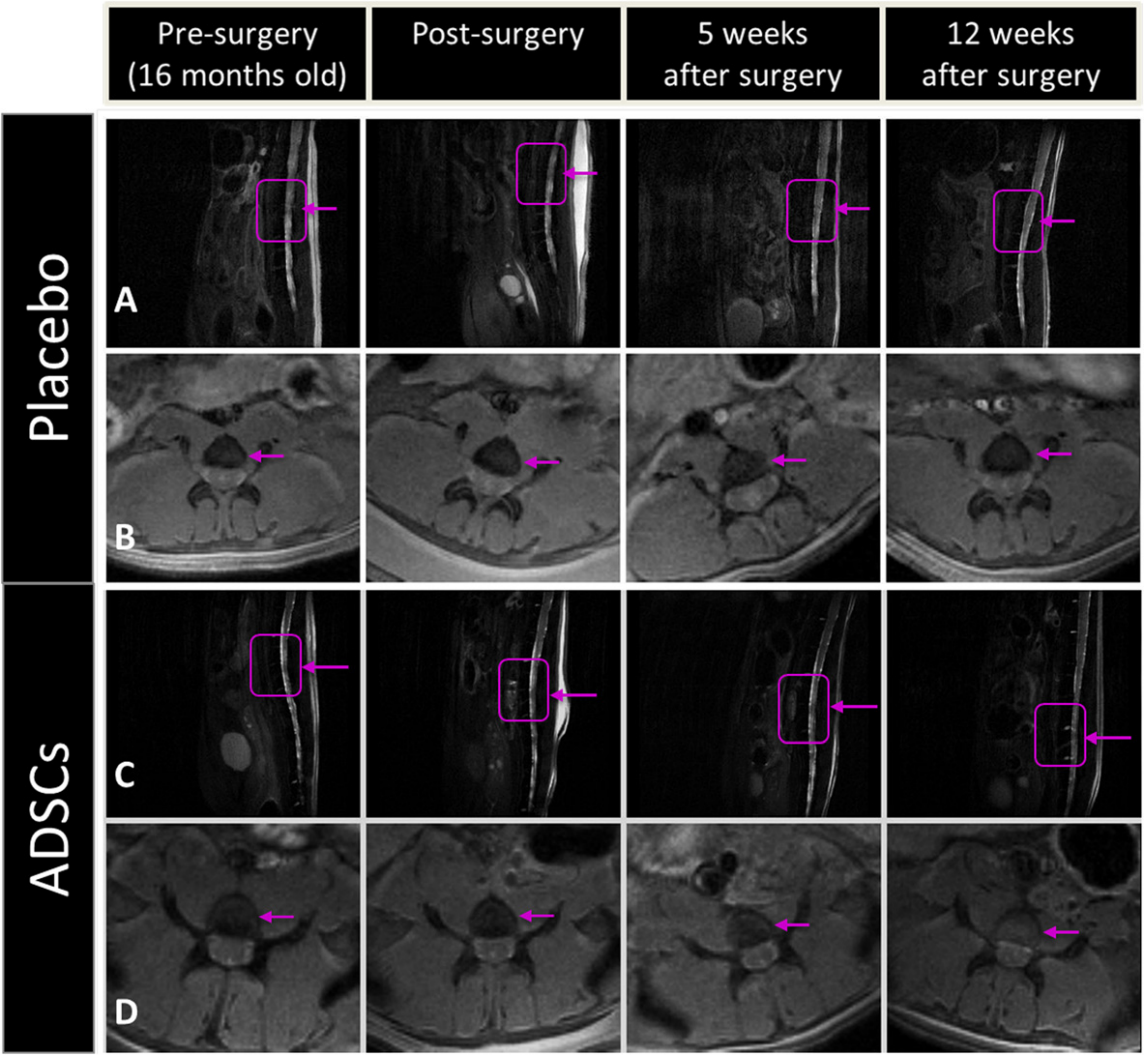
**Acquisitions of 7 Tesla magnetic resonance imaging on placebo and adipose mesenchymal stem cell (ADSC)-treated mice.** Sagittal T2-weighted **(A,C)** and axial T_1_-weighted images **(B,D)** of the lumbar tract are shown prior to (pre-surgery), 30 minutes after surgery (post-surgery), and 5 and 12 weeks after treatment for a representative mouse treated with placebo **(A,B)** or ADSC implant **(C,D)** of the degenerated disc. Violet boxes and arrows identify the spinal column level at vertebrae L1 to L2.

**Figure 4:**
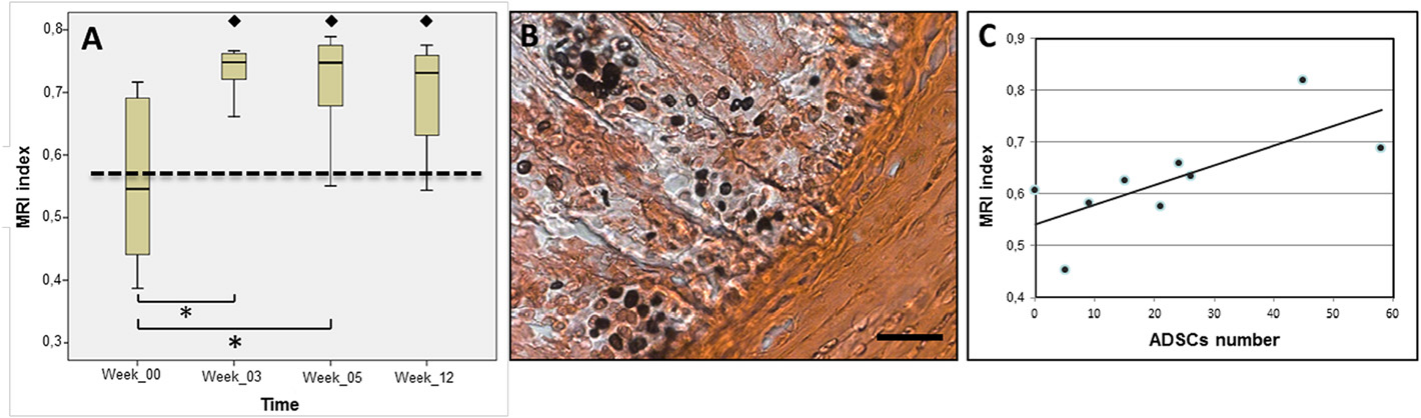
**Magnetic resonance imaging (MRI) index and adipose mesenchymal stem cell (ADSC) number in transplanted mice. (A)** MRI index in transplanted mice at 0 (pre-surgery), 3, 5 and 12 weeks after ADSC implant. Boxes refer to ADSC-implanted animals. *P <0.05, ADSC-implanted versus pre-surgery. The dotted line refers to placebo animals and represents the mean of all animals at different times after injection. ^♦^ *P* <0.05, ADSC-implanted versus placebo. **(B)** Representative intervertebral disc histological section of ADSC positivity to anti-HuNu and counterstaining with Safranin-O at 12 weeks after transplant. Scale bar, 100 μm. **(C)** Correlation between ADSC number and MRI intensity index at 12 weeks after transplant. Cell number was counted in a circular region of one mm^2^ from a representative slide of disc slab. The point corresponding to zero ADSCs, represents the mean of placebo MRI values.

Figure 3 shows sagittal T_2_-wi of a placebo and an ADSC-treated mouse pre-surgery, immediately post surgery (30 minutes) and 5 and 12 weeks post surgery. MRI revealed a visible increase in signal intensity within the discs of mice implanted with ADSCs (Figure 3C,D). On the contrary, placebo animals maintained low signal intensity along with time (Figure 3A,B). Indeed, in the placebo group, we observed that the MRI indexes were similar with time. In particular, this index was found to be 0.60 ± 0.067 and 0.57 ± 0.045 at time 0 (pre-surgery) and 12 weeks after injection, respectively, consistent with animal enrollment at 16 months, when degeneration was maximal. In contrast, in ADSC-treated mice the MRI index distribution was increased at 3, 5 and 12 weeks with respect to the baseline (pre-surgery) distribution, with statistical significance at 3 and 5 weeks (Figure 4A). In addition and of relevance, statistical analyses revealed a significant increase in the MRI index of the transplanted versus placebo mice at all investigated times, including 12 weeks (Figure 4A).

Twelve weeks after treatment, the slices of ADSC-implanted discs were immunopositive for both Safranin-O and anti-HuNu, which identify the ECM and human cells, respectively (Figure 4B).

The human cell number observed in the middle of the section of the cell-transplanted disc exhibited an appreciable correlation with the intensity index obtained by MRI examination of the corresponding mouse (Figure 4C). The correlation analysis was conducted in a group of eight implanted discs and the mean value of the placebo group was shown. The linear regression coefficient was *R* =0.73, with a positive slope.

### Histological analyses of IVD treated with ADSCs

**Figure 5:**
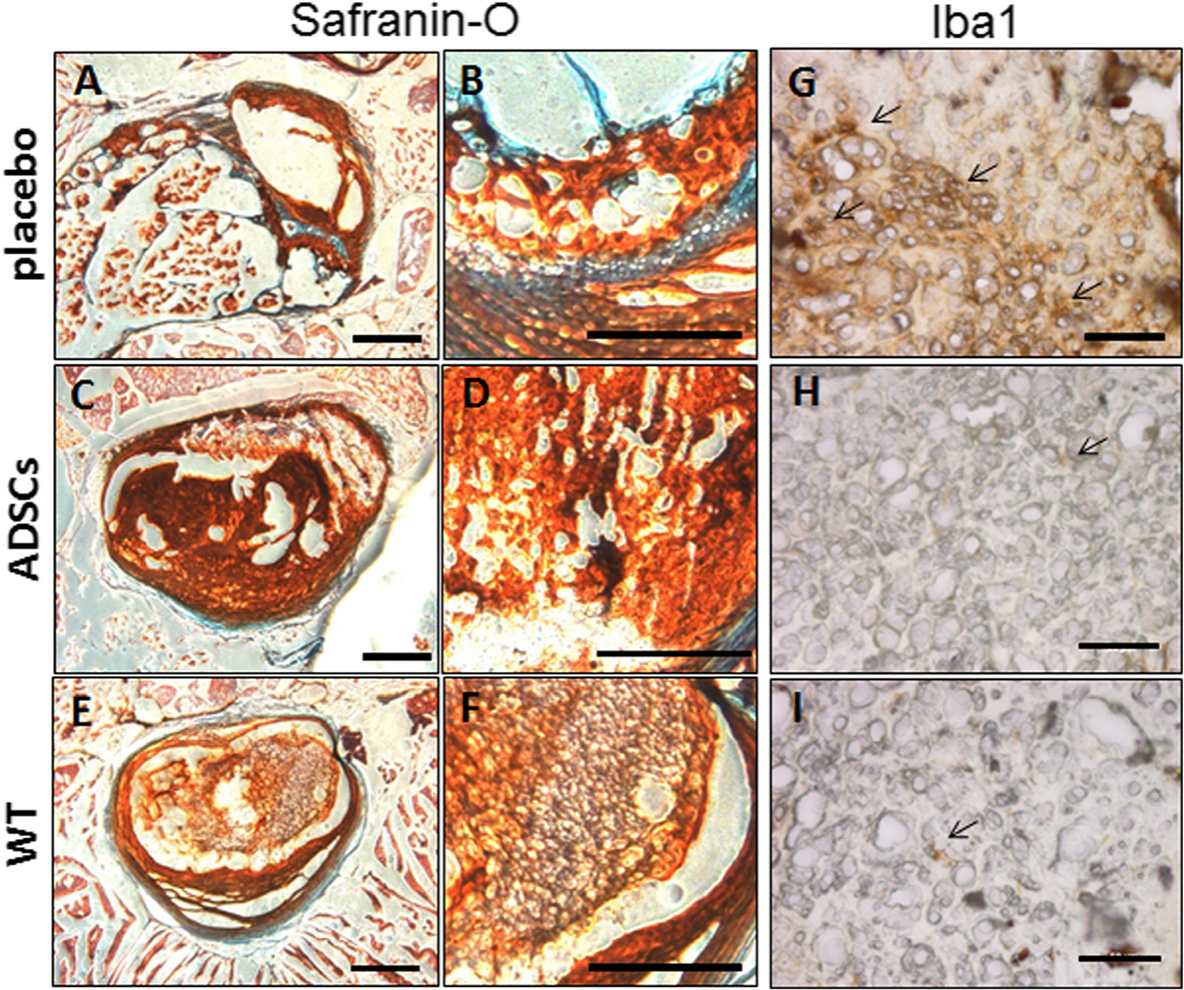
**Histological evaluation in placebo, transplanted and wild type (WT) mice.** Representative histological sections of Safranin-O **(A-F)** and Iba1 **(G-I)** immunostaining at the final follow-up period (12 weeks) from mice treated with placebo **(A,B,G)** or adipose mesenchymal stem cells **(C,D,H)**, and from WT mice **(E,F,I)**. Safranin-O staining is shown at two different magnifications (**A**,**C**,**E** and **B**,**D**,**F**, respectively). Scale bar: 500 μm **(A-F)** and 100 μm **(G-I)**. Iba1, ion calcium binding protein.

At 12 weeks post-transplant, all mice were sacrificed and IVD histology was performed. In the placebo group, all animals revealed a disordered pattern of fibrocartilage lamellas with a collapse of the inner annulus morphology (Figure 5A,B). In contrast, the discs of mice treated with ADSCs showed an improvement of the inner annulus structure and a recovery of the disc structure, evident after Safranin-O staining (Figure 5C,D), and appeared more similar to the WT group (Figure 5E,F) than to the placebo one. In addition, at high magnification, it was observed that the tissue architecture was more structured in ADSCs-treated mice than placebo ones, and again it was similar to that of WT mice.

ADSCs, placebo and WT groups were also investigated for the immunoinflammatory marker ion calcium binding protein (Iba1), which is specifically expressed in macrophages and activated microglia. In placebo sections, we observed an average of 45 to 50 positive cells per field (Figure 5G). Conversely, reactive cells in the ADSC group were by far less numerous (5 to 6 per field), and were characterized by a low intensity staining (Figure 5H). The number of positive cells in the ADSC group was found similar to that of WT samples (Figure 5I).

### Expression of HuNu, biglycan, aggrecan and Ki67 in mice treated with ADSCs and placebo

**Figure 6:**
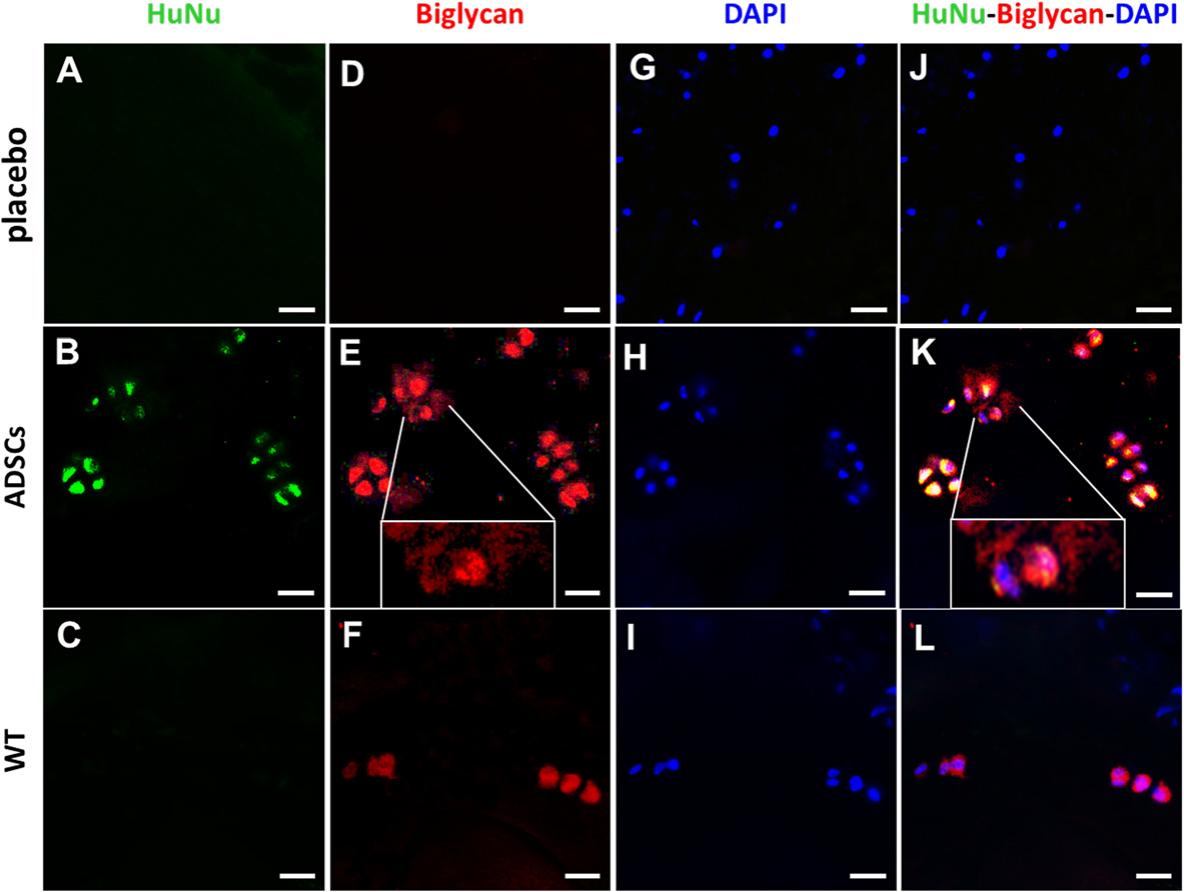
**Immunostaining of biglycan, 4',6-diamidino-2-phenylindole (DAPI) and human nuclei (HuNu) in the intervertebral disc (IVD).** Immunostaining of HuNu **(A-C)**, biglycan **(D-F)**, DAPI **(G-I)** and their colocalization **(J-L)** in IVDs are shown. The images are representative of placebo **(A,D,G,J)** and adipose mesenchymal stem cell (ADSC) mice **(B,E,H,K)** at 12 weeks from injection, and of wild type (WT) **(C,F,I,L)** animals at similar age. In the inserts of panel E and K, a magnification of biglycan immunostainings are shown. Scale bar, 50 μm.

**Figure 7:**
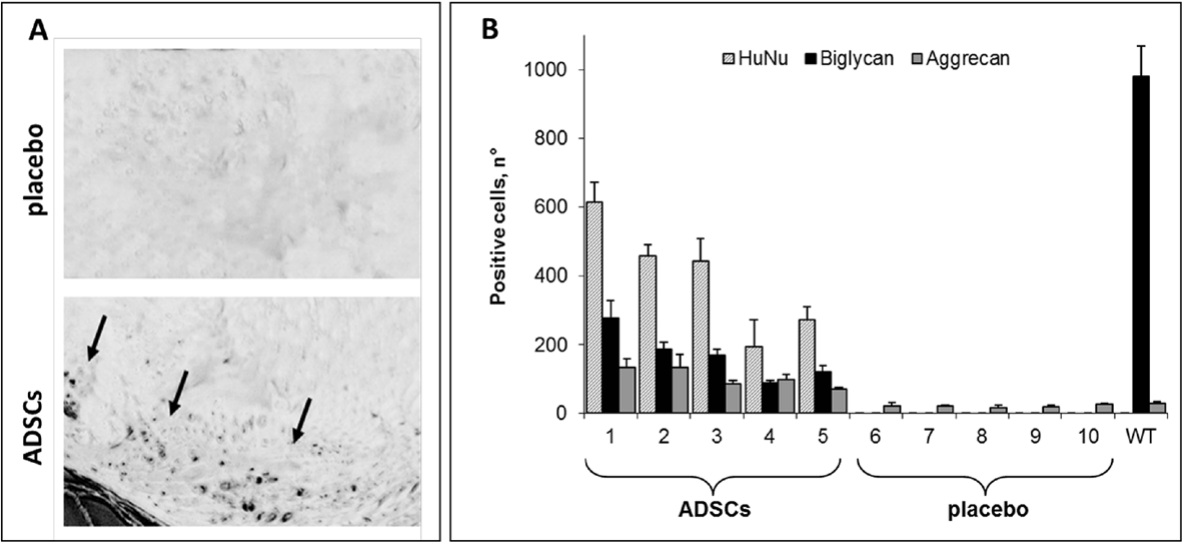
**Engraftment and co-localization of human nuclei (HuNu)-positive cells with biglycan. (A)** Representative images of HuNu immunostaining of a disc adjacent to the injected one (vertebrae L1 to L2) in placebo (upper panel) and adipose mesenchymal stem cell (ADSC)-implanted (lower panel) group are shown. Black arrows indicate the disc region were HuNu-positive cells are present. **(B)** The number of HuNu- (light gray), biglycan- (black) and aggrecan- (dark grey) positive cells in intervertebral discs from ADSC-transplanted and placebo groups are shown. Positive cells were measured in the whole discs of five individual animals transplanted with ADSCs (1 to 5), or injected with saline (6 to 10) at 12 weeks of treatment. Positive cells in five wild type (WT) animals of similar age are shown on the right. Data are the mean ± S.D.

We next analyzed HuNu (a marker for human cells) and biglycan expression by immunohistochemistry. At 12 weeks after transplantation, the staining with anti-HuNu in the WT and placebo groups was found negative (Figure 6A,C), whereas the ADSC-implanted mice showed positive expression (Figure 6B), indicating the presence of human cells. Interestingly, further analyses revealed that *Bgn*^−/0^ mice treated with ADSCs were also biglycan-positive (Figure 6E), as it occurs in the WT (Figure 6F). Although the biglycan immunopositive signal was found mainly localized intracellularly, this fluorescence was also detectable in the extracellular region (Figure 6E, insert), thus indicating that biglycan was also present in the ECM. By contrast, cells in placebo-treated *Bgn*^−/0^ mice were biglycan negative (Figure 6D). It was interesting to observe that biglycan-positive cells were also HuNu-positive (Figure 6K), suggesting that, once transplanted, human ADSCs may produce biglycan in mice lacking this capability. The analysis of HuNu expression also revealed that some human cells were present also in the adjacent disc (Figure 7A).

**Figure 8:**
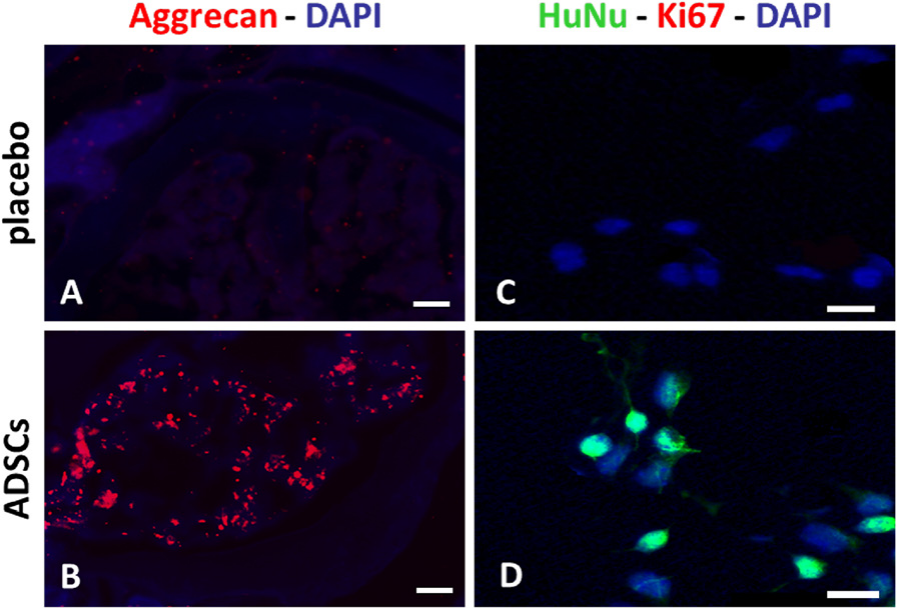
**Immunolocalization of aggrecan and human nuclei (HuNu) in placebo and adipose mesenchymal stem cell (ADSC) groups. (A,B)** Immuno-colocalization of aggrecan and 4',6-diamidino-2-phenylindole (DAPI) in placebo **(A)** and ADSC-implanted **(B)** groups. Scale bar, 200 μm. **(C,D)** Immunolocalization of Ki67, HuNu and DAPI in placebo group **(C)** and ADSC-implanted **(D)** groups. Scale bar, 50 μm.

We then evaluated the total number of cells positive for HuNu, biglycan and aggrecan in the whole discs. The results showed that HuNu-positive cells were present in all implanted discs from *Bgn*^−/0^ mice (Figure 7B, 1 to 5, light gray), and this number correlated with the count in a single histological slice (Figure 4C), indicating a diffuse localization of ADSCs in the discs. In all transplanted discs, biglycan- and aggrecan-positive cells were present, and accounted for 35 to 40% and 15 to 35% of Hu-Nu-positive cells, respectively (Figure 7B, 1 to 5, black and dark gray bars). In all placebo discs, immunopositivity for HuNu and biglycan was undetectable, whereas that for aggrecan, a critical component of ECM of IVD [27], was measurable at very low levels (Figure 7B, 6 to 10). The number of aggrecan-positive cells in the placebo group was found by far lower than that in the *Bgn*^−/0^ mice treated with ADSCs (Figures 7B and 8A,B).

In order to assess whether the ADSCs expressed aggrecan spontaneously, we performed immunohistochemistry on control cells and on cells growing in standard culture conditions, that is, in the absence of chondrogenic inducing factors. In both cases, we found that immunopositivity to aggrecan was undetectable in cultured ADSCs under normal expansion conditions (data not shown), indicating that they did not spontaneously express this large proteoglycan prior to implantation.

Finally, due to the significant presence of human ADSCs in transplanted mice, in order to ascertain whether ADSCs may undergo proliferation, we analyzed the expression of Ki67 at 12 weeks from ADSC injection. However, in *Bgn*^−/0^ mice at 12 weeks after implant, co-localization of HuNu and Ki67 staining was undetectable (Figure 8C,D).

## Discussion

Different mouse models have been proposed to study the physiological and pathophysiological behavior of degenerated IVD in spinal disorders. Numerous methods have been developed, including mechanical instability, mechanical compression, structural and chemical injury [28,29]. However, these models do not appear to entirely reflect disc degeneration, but rather they mirror post-surgery disc injury [30,31].

The *Bgn*^−/0^ mouse model exhibits many similarities to the human IVD pathophysiology, and despite not entirely reproducing it, it was reported to represent a valuable model for studying the time progression of IVD degeneration as a chronic and linear degenerative process *in vivo* [2]. Thus we considered the *Bgn*^−/0^ mouse as a suitable tool for our preliminary *in vivo* evaluation of the potential efficacy of ADSC implants as a therapeutic approach in progressive, degenerative disc disease.

In a previous study on *Bgn*^−/0^ mice, the degree of degeneration or deceleration of the disc degeneration process was evaluated by histological/immunochemical approach [2]. This type of evaluation of the IVD degeneration allows the identification of alterations in cell proliferation and ECM synthesis, but cannot provide information on the progression or arrest of the disease over time. Instead, changes in MRI signal intensity on T_2_-wi of IVDs, represents an important parameter for evaluating the degree of disc degeneration [32]. Indeed, a decrease in signal intensity of T_2_-weighted MRI images correlates with the IVD water content, which in turn is associated with reduction in nuclear proteoglycans, a main feature of disc degeneration [33]. Thus, in the case of disc regeneration, the high-signal intensity in the T_2_-wi is restored, reflecting restoration of proteoglycan and water content [34]. It is for these reasons that in the present investigation we first followed the time progression of IVD degeneration in *Bgn*^−/0^ mice by high resolution MRI, and then processed discs for histological analyses.

Our studies, performed by 7TMRI assessment and histological evaluation, confirmed that IVD of *Bgn*^−/0^ mice were in a more advanced degenerative state than those of WT mice at every age examined, and thus, the biglycan deficiency significantly accelerated disc degeneration. By 7TMRI assessment, we found that IVD degeneration was particularly severe in *Bgn*^−/0^ mice at 16 months of age. For this reason we started the cell therapy at this time.

Diverse therapeutic approaches have been proposed to halt or reverse IVD degeneration [30]. These include treatments with anabolic growth factors [35] and, more recently, cell therapy using either intradiscal administration of NP-derived cells, or MSCs of different tissue origin [16,17,36]. In this context, the use of ADSCs was recently proposed as a potential and effective therapeutic tool for the treatment of IVD degeneration [37]. Prompted by this evidence, we used human ADSCs isolated without enzymatic digestion from abdominal adipose tissue specimens. These cells in culture exhibit proliferative activity, maintain typical mesenchymal markers, as well as differentiation potential [18].

Some evidence of IVD regeneration in an autologous ADSC-injected rabbit model has been previously provided [38]. The present study shows for the first time that ADSC transplantation is beneficial in ameliorating degeneration in a chronic model of IVD, as demonstrated by MRI, histological and immunofluorescence findings.

In particular, the ameliorative effect of ADSCs on IVD degeneration was evident as MRI index values at all investigated times, with significant differences when compared to the pre-operative value. Of relevance, the MRI index values of the ADSC-treated group were significantly higher than those of the placebo group at all investigated times. Moreover, we found that the MRI intensity index correlated with the number of cells found in the implanted discs, supportive of an increment in water content and proteoglycans synthesis in the presence of ADSCs. The histological assessment by Safranin-O staining confirmed that ADSC implants improved the structure of IVD in *Bgn*^−/0^ mice. Indeed, we observed a recovery of disc structure, supporting that neosynthesis of ECM components occurred in transplanted discs. However, it is important to note that transplanted cells may also have transient paracrine effects, and their survival in the site of injection could not be the only one relevant aspect involved in disc regeneration [37].

Although the beneficial effects of ADSC transplantation were already significant after 3 weeks, these effects appeared not to progress, but rather to be maintained along with time. Notwithstanding the ameliorative effects of ADSC transplantation on disc degeneration, the apparent lack of increased beneficial effects over longer periods of time may indicate that a unique treatment could not be sufficient for optimal therapy, and that different treatments (in terms of schedules and/or transplanted cell number) could be more effective than the one used for longer periods. Further experiments are needed to clarify if serial transplants, and/or a higher number of implanted cells, as well as ADSC injections at earlier degeneration times could be effective in ameliorating the recovery process of IVD degeneration.

Immunofluorescence analyses performed with antibodies against human HuNu, Ki67, biglycan, and aggrecan provided additional important results, which we subsequently discuss here. Of critical importance, our data revealed the presence of HuNu positive cells, that is, of human cells, in transplanted IVDs all over time, and up to 12 weeks post-transplant. Thus, it emerges that, after implantation into the degenerated IVDs of biglycan-deficient mice, some human ADSCs can survive for at least 12 weeks in the peculiar microenvironment of the IVD, where hypoxia, low nutrition, acidic pH, mechanical loading, endogenous proteinases, and cytokines [39] would not favor exogenous ADSC survival. We also observed some positivity for anti-HuNu also in the adjacent discs, possibly due to the leakage of cells during injection.

However, notwithstanding the presence of ADSCs in IVDs, at 12 weeks we were unable to find any evidence of cell division, as the proliferation marker Ki67 was undetectable at this time. However, taking into account that 12 weeks is a prolonged time after transplantation, we cannot exclude that ADSC proliferation may occur at earlier times. As indirect support of this hypothesis, histological images revealed that the implanted cells are present as tight clusters of cells, which often indicates a sign of cell division. Thus, ADSCs might have undergone cell division in the early weeks and then stopped in the later weeks.

Additional important evidence provided by our study is that a relevant percentage of ADSCs transplanted on IVD of *Bgn*^−/0^ mice showed positivity to anti-human biglycan, and also to aggrecan, supporting the concept that a differentiation process in ADSCs has occurred at 12 weeks from transplant. This is in agreement with previous studies on cultured cells, demonstrating the stem cell population within the adipose stromal compartment in human processed lipoaspirate can differentiate toward the osteogenic, adipogenic, myogenic, and chondrogenic lineages, and that they express biglycan and aggrecan in chondrogenic differentiation conditions [40]. Notably, although human mesenchymal stem cells have been reported to spontaneously express some ECM proteins such as cartilage oligomeric matrix protein [41], we obtained evidence that ADSCs did not spontaneously express aggrecan when cultured in the absence of chondrogenic inducing factors, that is, in the normal expansion conditions we used prior to implantation. Therefore, it emerges that human ADSCs are multipotential stem cells capable of undergoing chondrogenic differentiation *in vivo*, and it is suggested that the diseased disc environment is able to induce the expression of both biglycan and the cartilage specific proteoglycan aggrecan. Whereas both proteins appear to be *de novo* produced by implanted ADSCs, as placebo-treated mice showed some positivity to anti-human aggrecan antibody, we cannot exclude that ADSCs treatment may stimulate an endogenous production of aggrecan too, by a paracrine effect of the cells in the disc.

Overall, it is tempting to speculate that, once implanted into IVDs, ADSCs initially may undergo cell division and forms tight clusters, and subsequently, stimulated by the local microenvironment, close-fitting clusters of ADSCs undergo chondrogenic differentiation. In agreement, cell density was reported to be crucial for chondrogenic differentiation, pellet mass cultures being essential for the chondrogenic differentiation of MSCs [42].

Two further important findings of our study deserve comments. First, it is worth noting that the ameliorative effect of ADSC implant on disc degeneration was observed in old animals and these had severe disc damage. This underlies the potential of ADSC implant as effective in our *in vivo* mouse model. Second, during the study period, we did not observe any host-graft rejection, further supporting the effectiveness of ADSC implants in IVDs. In this context, the survival of IVD-implanted ADSCs may be favored by the fact that the IVD represents a privileged implantation site from the immunological point of view, mesenchymal stem cells derived from different adult tissues showing advantages from these immunosuppressive effects [43-45].

## Conclusion

In conclusion, our study shows for the first time that the ADSC treatment can be beneficial *in vivo* in a mouse model of spontaneous IVD degeneration. ADSCs exert their effects at various levels, overall positively influencing the degeneration process and finally exhibiting long-term engraftment and tissue recovery. ADSCs appear effective in IVD degeneration by providing exogenous biglycan, and simultaneously stimulating the endogenous production of aggrecan, both these proteoglycans being crucial in re-establishing the IVD matrix composition closer to the normal one [46].

Overall, results obtained in this study provide preliminary *in vivo* evidence that ADSC transplants may represent an effective treatment for degenerative disc disease in humans. Although the *Bgn*^−/0^ mouse is a valuable model for investigating disc degeneration [2], it is worth noting that this model does not entirely reproduce the human disc pathophysiology. Future investigations in different models such as non-human primate models are prompted by this research, and will hopefully confirm the validity of the proposed therapy.

## Abbreviations

7TMRI: 7-Tesla magnetic resonance imaging
ADSC: Human-derived adipose mesenchymal stem cell
AF: anulus fibrosus
*Bgn*^−/0^: biglycan-deficient mice
DAPI: 4',6-diamidino-2-phenylindole
DMEM: Dulbecco's modified Eagle's medium
D-PBS: Dulbecco’s phosphate-buffered saline
ECM: extracellular matrix
EGF: epidermal growth factor
ET: echo time
FACS: fluorescence-activated cell sorting
FGF2: basic fibroblast growth factor 2
FBS: fetal bovine serum
HuNu: human nuclei
IVD: intervertebral disc
MRI: magnetic resonance imaging
MSC: mesenchymal stem cell
NA: number of averages
NP: nucleus pulposus
PFA: paraformaldehyde
RARE: rapid acquisition with relaxation enhancement
SCM: stem cell medium
SLRPs: small leucine repeat proteoglycans
ST: slice thickness
T_1_-wi and T_2_-wi: T_1_ and T_2_-weighted images
TR: repetition time
WT: wild type
